# Method for Calculating the Bending Stiffness of Honeycomb Paperboard

**DOI:** 10.3390/ma17040878

**Published:** 2024-02-14

**Authors:** Gabriela Kmita-Fudalej, Zbigniew Kołakowski, Włodzimierz Szewczyk

**Affiliations:** 1 Centre of Papermaking and Printing, Lodz University of Technology, Wólczańska Str. 221, 93-005 Lodz, Poland; wlodzimierz.szewczyk@p.lodz.pl; 2 Department of Strength of Materials, Lodz University of Technology, Stefanowskiego Str. 1/15, 90-537 Lodz, Poland; zbigniew.kolakowski@p.lodz.pl

**Keywords:** honeycomb paperboard, cellular paperboard, bending stiffness, local buckling, mechanical properties

## Abstract

The article presents continued considerations presented in a prior publication on the development of a model for calculating the bending stiffness *BS* of cellular honeycomb paperboards, applying the strength properties of paper raw materials used for the production of paperboard and the geometric parameters of cellular board. The results of *BS* calculations obtained by using the analytical model presented in the prior publication were significantly overestimated in relation to the value obtained by measurements. The calculation error in relation to the measurement value for the tested group of paperboards in the case of bending stiffness in the machine direction *MD* was within the range from 23% to 116%, and the average error was 65%, while in the cross direction *CD,* it was within the range from 2% to 54%, and the average error was 31%. The calculation model proposed in this work based on the physical properties of cellular paperboard reduces the error values for bending stiffness in both the machine and cross directions. The value of the average error for both main directions in the paperboard plane was 10%. The method enables more accurate determination of *BS* in the machine direction *MD* and in the cross direction *CD* at the paperboard design stage. In order to validate the proposed analytical model, the calculation results were compared with the results of *BS* laboratory measurements performed using the four-point bending method and, in order to expand the group of tested paperboards, with the measurement results presented in the prior article for cardboards with different raw material composition and different geometric parameters.

## 1. Introduction

The production process of cellular structure paper core was started by Hans Heilbrun at the Heilbrun and Pinner paper factory in Halle, Germany, and then, in 1901, he patented the honeycomb production process [1,2,3].

For many years, multi-layer paper materials, owing to the use of ecological, biodegradable, and recyclable raw materials for their production, have become very popular in various industry branches [4,5]. Honeycomb paperboard and cores are applied in many products and are gradually replacing products made of wood, plastics, or aluminum due to lower production costs and lower specific gravity. Other advantages of honeycomb paperboard are high strength and excellent energy absorption properties, as well as good insulating, thermal, and acoustic properties [6,7]. Honeycomb cores are present in our everyday life. They are used as fillings for doors, furniture countertops, furniture boards, partition walls in construction, and multi-layer sandwich structures in the aviation and automotive industries [8,9,10,11]. The production and use of paper-filled honeycomb panels in the furniture industry are developing rapidly in Europe [12]. The demand for lighter furniture elements is increasing, which contributes to lower transport costs and easier assembly, while reducing formaldehyde emissions, which is also an important issue in the modern world [13]. Honeycomb cores are also used as fillings for school boards, and ping-pong and billiard tables. There are many articles in the literature about experimental, analytical, and numerical studies of paper products. Methods for measuring the bending stiffness of cellular structures are widely described by Tom Bitzer [14]. For *BS* measurements, he uses the three-point and four-point bending method. He uses instruments of various designs for testing. The three-point loading method uses supports with a rigid structure without the possibility of rotation and adjustment of the supports to the sample deformation during the measurement, which contributes to the movement of the contact line between the supports and the tested sample and the change in the measurement length. The four-point bending method uses supports with the possibility of rotation relative to an axis parallel to the tested sample width and adjustment to the sample deflection during measurement, which ensures constant measurement length.

Publications [4,15,16] describe *BS* testing methods for panels consisting of a honeycomb core combined with thin furniture boards. Bending stiffness tests of a paper honeycomb core fixed between two furniture panels were performed according to EN 310:1993 “Wood-based panels—Determination of modulus of elasticity in bending and of bending strength”. Supports of a circular cross-section were used, which well reproduce the load model adopted for *BS* calculations and can be used to test materials with a very hard surface.

Smardzewski et al. [17] conducted research to determine the effect of the rectangular cell shape of the paper core on the mechanical properties of three-layer furniture panels and compared them with the properties of a panel with hexagonal cells of the paper core with the same geometric parameters. The measurements were made for both main directions in the panel plane—machine direction and cross direction. The tests showed that the rectangular cells of the core increased the stiffness of the furniture panels in the cross direction and, at the same time, decreased this stiffness in the machine direction.

The behavior of paper honeycomb panels subjected to bending and compressive loads was investigated in article [18] where the authors apply finite element modeling using a simplified model of a honeycomb core with slight displacements to evaluate the deformations and stresses in the component materials under bending and crushing loads.

Guo Y. in the article [19] investigated the shock absorption and vibration transmission properties of honeycomb paperboard of different thicknesses using a series of experimental tests on a shock machine and a vibrating table.

Gao S. and Wang B.Z. [20,21] examined laminates composed of two honeycomb paperboards by means of a finite element model. In the models, they used different shapes of cells: a regular hexagon and a rhombus. The numerical results show that the compression strength of the double-layer honeycomb is better than that of the single-layer honeycomb.

Wang and Yao [22] by means of experiments and FEM studied the impact load capacity and energy absorption capacity of honeycomb paperboard with different ratios of cell wall thickness to its length for different cardboard moisture contents. The effect of honeycomb cell size and cell wall thickness on the crushing strength of kraft paper honeycomb core was also numerically analyzed by Kadir et al. [23].

The authors of publications [22,24] analyzed the effect of geometric parameters of honeycomb paperboard on cushioning and shock absorption during free fall. The tests have shown that the thickness and length of the wall of the hexagonal cell of a honeycomb core have a huge impact on its shock-absorbing properties. Reducing the cell size of the cellular paperboard core improves the energy absorption capacity, and the thickness of the paper honeycomb core has a variable effect on the shock-absorbing properties. Chen and Yan [25] analyzed the effect of the thickness of a honeycomb core made of kraft paper and flat layers made of MDF board on the stiffness of a sandwich panel. They developed finite element models for the resulting sandwich panels. The studies have shown that reducing the ratio of the thickness of the paper core to the thickness of the flat layer results in an increase in the elastic modulus and shear modulus of sandwich panels. Chen et al. [26] conducted research on the flexural creep of sandwich panels with a honeycomb core made of kraft paper with flat layers of wood composite. The sandwich panels contained different types of core and flat layer materials, as well as different core and flat layer thicknesses. The creep deflection during bending was measured as a function of time for each type of sandwich panel. The results showed that the flexural creep of the sandwich panel is influenced by the shape of the honeycomb core cell, the thickness of the core and flat layers, and the type of lining material. Chen et al. [27] also conducted research on lightweight multi-layer panels with different honeycomb core structures made of paper and composite wood lining. Using experimental research and finite element methods, the authors presented the impact of honeycomb design parameters as well as core and lining material properties on the mechanical properties of lightweight laminated panels. Many works deal with theoretical considerations in terms of the analysis of the stability of composite structures [28,29,30,31].

The analytical model presented in the previous publication [1] did not consider the local buckling of flat layers subjected to compression during *BS* measurement and showed how much this imperfection of the honeycomb paperboard affects the bending stiffness. In addition, the phenomenon of local buckling of the flat layer was generally ignored in the research conducted so far.

The developed model for calculating *BS* in both main directions in honeycomb paperboard considers the local buckling of the flat layer subjected to compression during *BS* measurement.

According to the authors, this is a novelty of the proposed method of calculating *BS*.

The method enables more accurate determination of BS in the machine direction *MD* and in the cross direction *CD* at the paperboard design stage.

The greatest advantage of the presented calculation method is the ability to predict bending stiffness based on the mechanical properties of the papers used to produce paperboard and the geometric parameters of the paperboard. Thanks to the ability to predict bending stiffness, it is possible to select appropriate fibrous raw materials and geometric parameters of the honeycomb paperboard before producing paperboard with the required stiffness, so that the product meets the customer’s requirements and is economical in production.

## 2. Materials and Methods

**Figure 1:**
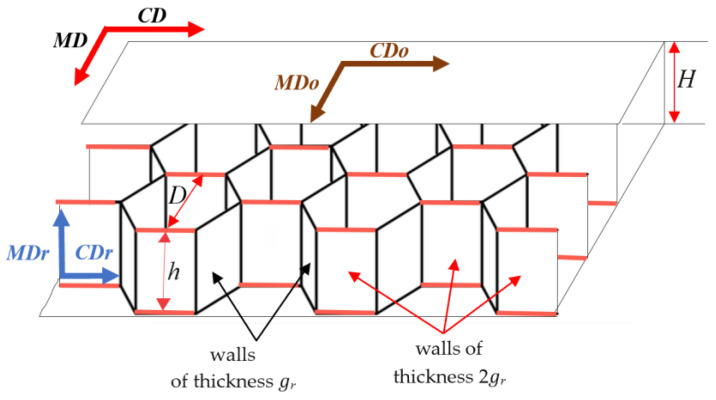
Geometric parameters of cellular paperboard and main directions in the paperboard plane: *D*—diameter of a circle inscribed in a regular hexagon (referred to as the cell size), *h*—core height, *H*—paperboard thickness, *g_r_*—thickness of the core paper.

The subject of the analysis was cellular honeycomb paperboards made of various fibrous raw materials and with various geometric parameters, the construction of which is depicted in Figure 1. Cellular paperboard is a laminate made of a core connected by an adhesive joint to two flat layers. The honeycomb core has a characteristic structure consisting of adjacent spatial cells in the shape of a regular hexagon. In its structure, we can distinguish the walls of a hexagonal cell with a single and double thickness of the paper from which it is made. The distribution of mechanical properties of honeycomb paperboard, similarly, to corrugated boards and papers for the production of corrugated boards, is a distribution characteristic of orthotropic bodies. In the plane of cellular paperboard, there are two main directions of orthotropy: the machine direction coinciding with the MD direction of paperboard production, and the cross-direction *CD* perpendicular to the machine direction. As shown in Figure 1, the paperboard main directions *CD* and *MD* coincide with the main directions of paper used for the production of paperboard flat layers *CD_O_* and *MD_O_*, respectively. In the case of a paper core, the machine direction of the paper used for the core *MDr* is parallel to the core height, and the cross direction *CDr* is perpendicular to the core height.

Eight cellular honeycomb paperboards made of various fibrous raw materials and with different geometric parameters were subjected to bending stiffness *BS* measurements. 

Table 1 presents the raw material composition of honeycomb paperboards, and Table 2 presents the geometric parameters of cellular paperboards.

### 2.1. Experimental Research

**Figure 2:**
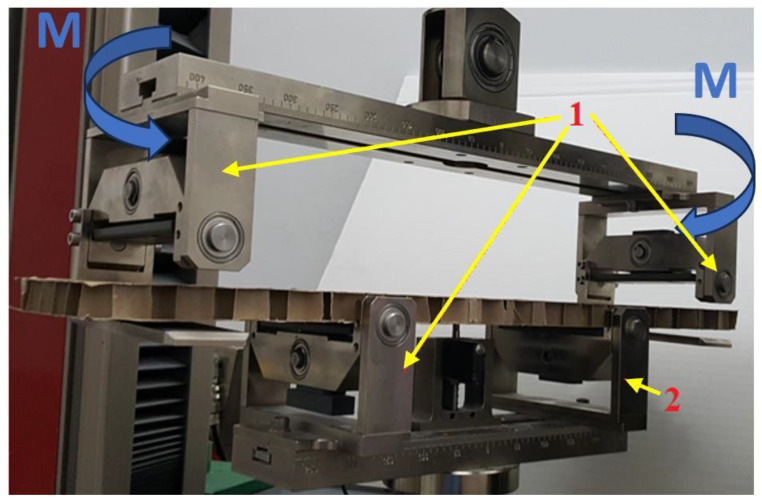
Tool for measuring bending stiffness: 1—support with two degrees of freedom, 2—support with one degree of freedom, M—bending moment.

The measurement of bending stiffness was carried out in two main directions in the plane of the paperboard—machine *BS_MD_* and cross direction *BS_CD_* in accordance with the PN-EN 5628:1995 standard, ”Paper and paperboard—Determination of bending stiffness by static methods—General principles”. The test was performed on a Zwick Tensile Machine model Z010 from Zwick Roell Group (Ulm, Germany) equipped with appropriate equipment for measuring *BS* using the 4-point bending method. Samples with dimensions of 500 mm × 100 mm were bent with a moment M lying in a plane perpendicular to the outer plane of the paperboard and parallel to the longer side of the sample shown in Figure 2.

**Figure 3:**
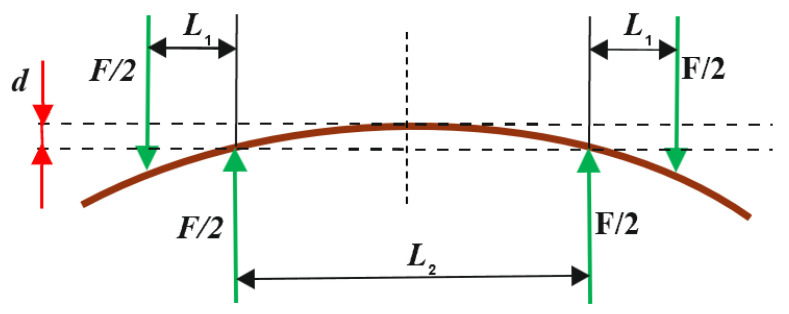
Loading diagram applied in *BS* measurement tests using the four-point loading method.

The distribution of forces acting on the sample is shown in Figure 3. 

The *BS* measuring device had supports with a rectangular cross-section, 30 mm wide and 103 mm long. The distance between the supports and applied forces amounted to $L_{2}=200$ mm and $2L_{1}+L_{2}=400$ mm, respectively.

Bending stiffness was calculated from the following Formula (1) using the designations given in Figure 2 according to PN—ISO 5628:1995 standard, “Paper and paperboard—Determination of bending stiffness by static methods—General principles”:

(1) $$BS=\frac{F\;L_{1}L_{2}^{2}}{16db}\;\;\left[Nm\right]$$

where

*F*—force loading the paperboard in the bending test, N;

*L*_1_, *L*_2_—distances between supports, m;

*d*—deflection of the tested sample caused by the force *F*, m;

*b*—sample width, m.

The applied speed of movable supports was 10 mm/min. A detailed description of the applied instrument, measurement methodology, and parameters is described in the publication [1]. The final result is given as the average value of 10 measurements separately for the machine direction *BS_MD_* and the cross direction *BS_CD_*.

Measurements of the thickness of paperboards and papers were also performed, and Young’s modulus was determined for the papers from which the tested paperboards were made. A Handyworth caliper with electronic reading, model MC0901, was used to measure the thickness of the honeycomb paperboards. Twenty thickness measurements were made for each honeycomb paperboard. The basic principle of thickness measurement was to properly set the caliper so that its measuring part covered at least two double walls of the cellular paperboard core. This measurement method reduced the impact of too high pressure on the measurement result because double walls have much higher stiffness.

The paper thickness was measured according to PN-EN ISO 534:2012, “Paper and paperboard—Determination of thickness, apparent density and specific volume”. The measurements were made by means of a micrometer equipped with two parallel pressure plates with an area of 2 cm^2^ each, using a pressure of 100 ± 10 kPa, which was exerted on the tested paper by the surfaces of the plates. 20 measurements were made for each paper. The thickness of papers and paperboards is given as an average value of 20 measurements.

The Young’s modulus was determined on the basis of a paper tensile test at a constant tensile speed according to PN-EN ISO 1924-2:2010 standard, “Paper and cardboard—Determination of properties under tensile forces—Part 2: Test at a constant tensile speed (20 mm/min)”. The width of the samples was 15 mm and the length of the samples was 180 mm. The tensile testing was performed at a constant speed of 20 mm/min. Young’s modulus was measured in two main directions in the *CD* and *MD* paper plane. The measurements were made by a universal testing machine Zwick Tensile Machine model Z010 from Zwick Roell Group (Ulm, Germany).

### 2.2. Model for Calculating the Cellular Paperboard BS

The paper proposes the determination of the bending stiffness *BS* based on the classical laminated plate theory (CLPT) and generally known material strength formulas [32,33]. Detailed formulas are given in Appendix A.

#### 2.2.1. Method for Calculating the Stiffness of Flat Layers of Cellular Paperboard

**Figure A1:**
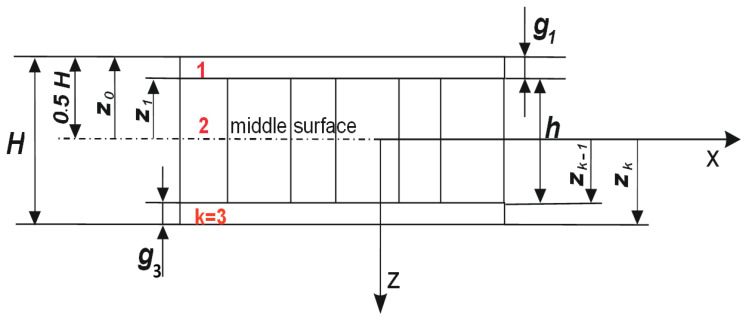
Geometry of the *k*-th layer.

In the first approach, the bending stiffness of the flat layers was calculated in both main directions, disregarding the core of the cellular paperboard (i.e., layer k = 2 Appendix A, Figure A1); i.e., the stiffnesses *A*, *B*, and *D* were disregarded, which means that the following was assumed:

(2) $$E_{12}=E_{22}=0\;$$

**Figure 4:**
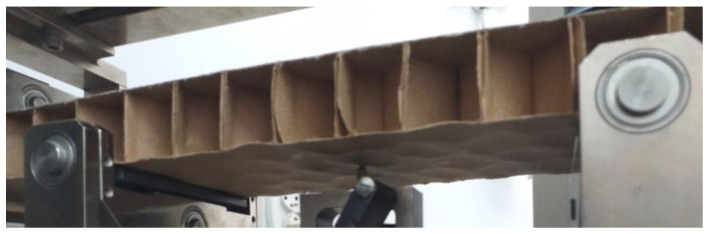
View of buckling of compressed flat surface of cellular paperboard [1].

During the experiment carried out to determine stiffness, the phenomenon of buckling of the paperboard flat layer marked as layer k = 3 (Appendix A, Figure A1) was observed, as shown in Figure 4. This was taken into account in the proposed method by introducing a material stiffness correction factor ψ, i.e., Young’s modulus correction *E*_13_ and *E*_23_.

In the tested cellular paperboards, the flat layers were identical $g_{1}=g_{3}=g_{o}$, i.e., *E*_11_ = *E*_13_ = *E_oMD_* and *E*_21_ = *E*_23_ = *E_oCD_*.

The final formulas resulting from the proposed method according to Formulas (A1), (A3), (A4), (A5), (A12), and (A15) presented in Appendix A and the above assumption defined by Formula (2) are listed below.

The component stiffnesses determined for the machine direction *MD* take the form:

(3) $$A_{11}=E_{oMD}g_{o}\left(1+\psi_{1}\right)$$

(4) $$B_{11}=\frac{E_{oMD}\left(g_{o}^{2}-Hg_{o}\right)\left(1-\psi_{1}\right)}{2}$$

(5) $$D_{11}=\frac{E_{oMD}\left(0.75H^{2}g_{o}-1.5Hg_{o}^{2}+g_{o}^{3}\right)\left(1+\psi_{1}\right)}{3}\approx \frac{E_{oMD}\left(0.75H^{2}g_{o}-1.5Hg_{o}^{2}\right)\left(1+\psi_{1}\right)}{3}$$

where

*E_oMD_*—Young’s modulus of paper used to produce flat layers of cellular paperboard in the machine direction *MD*;

$g_{o}—$thickness of paper used to produce flat layers of cellular paperboard;

$\psi_{1}—$correction factor of the Young’s modulus of paper used to produce flat layers of cellular paperboard in the machine direction *MD*.

$$0<\psi_{1}\leq 1.0$$

- when $g_{o}/g_{r}$ ≥ 1.35 then $\psi_{1}$ = 1.00;
- when 1.35 > $g_{o}/g_{r}\;$≥ 1.15 then $\psi_{1}$ = 0.70;
- when 1.15 > $g_{o}/g_{r}$ ≥ 1.00 then $\psi_{1}$ = 0.60;
- when $\;g_{o}/g_{r}$ < 1.00 then $\psi_{1}$ = 0.50.

The correction factor of the Young’s modulus of paper used to produce flat layers of cellular paperboard in the machine direction *MD*, $\psi_{1},$ takes into account the elastic impact of the honeycomb middle layer (core) in the *MD* direction on the flat layers. This elastic effect depends on the dimensions of the honeycomb structure and the thickness of the flat layer.

The value of the $\psi_{1}$ coefficient considers the local buckling of the skin layers, which is the novelty of this article. The values of $\psi_{1}$ were adopted taking into account the theoretical basis of elastic support of the flat layers, validated by the results of experimental tests.

**Figure 5:**
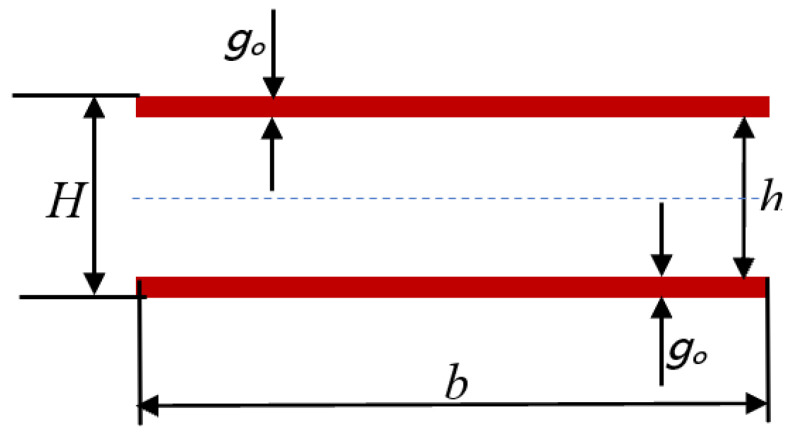
Diagram of a simplified paperboard cross-section: *H*—paperboard thickness, $g_{o}—$thickness of flat layer papers, *b*—sample width, ℎ—core height.

The bending stiffness of cellular paperboard flat layers in the *BS*_1_ machine direction, taking into account the parameters according to Figure 5, is calculated from Formula (A12) included in Appendix A, which takes the form:

(6) $$BS_{1}=D_{11}^{*}b=D_{11}\alpha_{1}b=D_{11}\left[1-\frac{B_{11}^{2}}{A_{11}D_{11}}\right]b$$

In the case of paper materials, the bending stiffness is related to the sample width *b*; i.e., the formula for bending stiffness of linings in the machine direction *BS_oMD_* takes the form:

(7) $$BS_{oMD}=\frac{BS_{1}}{b}=D_{11}\left[1-\frac{B_{11}^{2}}{A_{11}D_{11}}\right]$$

In the cross direction *CD*, the component stiffnesses take the form:

(8) $$A_{22}=E_{oCD}g_{o}\left(1+\psi_{2}\right)$$

(9) $$B_{22}=\frac{E_{oCD}\left(g_{o}^{2}-Hg_{o}\right)\left(1-\psi_{2}\right)}{2}$$

(10) $$D_{22}=\frac{E_{oCD}\left(0.75H^{2}g_{o}-1.5Hg_{o}^{2}+g_{o}^{3}\right)\left(1+\psi_{2}\right)}{3}\approx \;\frac{E_{oCD}\left(0.75H^{2}g_{o}-1.5Hg_{o}^{2}\right)\left(1+\psi_{2}\right)}{3}$$

where

*E_oCD_*—Young’s modulus of paper used to produce flat layers of cellular paperboard

in the cross direction *CD*; 

$g_{o}—$thickness of paper used to produce flat layers of cellular paperboard;

$\psi_{2}—$correction factor of the Young’s modulus of paper used to produce flat layers of cellular paperboard in the cross direction *CD*.

$$0<\psi_{2}\leq 1.0$$

- when $g_{o}/g_{r}$ ≥ 1.35 then $\psi_{2}$ = 1.00;
- when 1.35 > $g_{o}/g_{r}\;$≥ 1.15 then $\psi_{2}$ = 0.90;
- when 1.15 > $g_{o}/g_{r}$ ≥ 1.00 then $\psi_{2}$ = 0.80;
- when $\;g_{o}/g_{r}$ < 1.00 then $\psi_{2}$ = 0.60.

The correction factor of the Young’s modulus of paper used to produce flat layers of cellular paperboard in the *CD* direction $\psi_{2}$, similarly to the coefficient $\psi_{1}$, takes into account the elastic impact of the middle layer (core) in the *CD* direction on the flat layers. The values of $\psi_{2}$ were selected similarly to for *MD* direction. 

The values of the coefficients $\psi_{1}$ and $\psi_{2}$ are different due to different models of flat layers reinforcement in the *MD* and *CD* directions, which is discussed in detail in Section 2.2.2.

The stiffness of cellular paperboard flat layers in the cross direction *BS*_2_ is calculated from Formula (A15) included in Appendix A, taking into account the parameters according to Figure 5, which takes the form:

(11) $$BS_{2}=D_{22}^{*}\;b=D_{22}\alpha_{2}b=D_{22}\left[1-\frac{B_{22}^{2}}{A_{22}D_{22}}\right]b$$

Relating *BS*_2_ to the sample width, the bending stiffness of the linings in the cross direction *BS_oCD_* is calculated from the formula:

(12) $$BS_{oCD}=\frac{BS_{2}}{b}=D_{22}\left[1-\frac{B_{22}^{2}}{A_{22}D_{22}}\right]$$

#### 2.2.2. Method for Calculating the Stiffness of Cellular Paperboard Core

**Figure 6:**
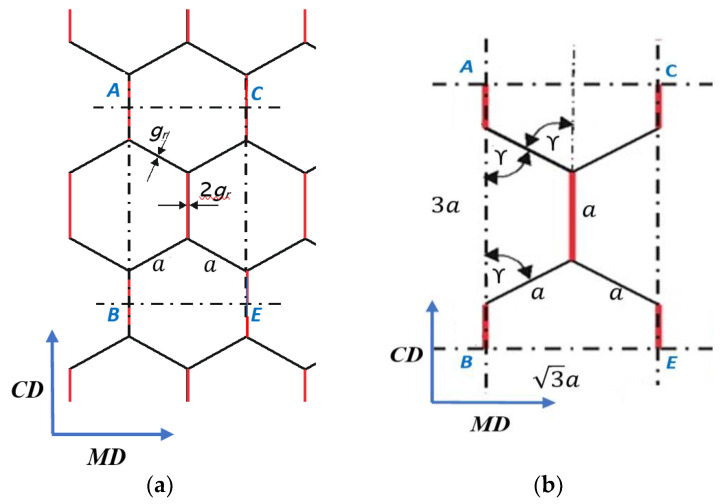
Periodic cell: (**a**) ACBE cell isolated from a paperboard core, (**b**) dimensions of the periodic cell, *a*—length of the side of the hexagonal cell, angle γ = 60°, *g_r_*—single wall thickness, 2*g_r_*—double wall thickness.

It was assumed that the calculation model of the stiffness of the paper core in both machine and cross directions referred to the *ACBE* periodic cell (Figure 6a) isolated from the core structure with dimensions in the *MD* direction $\sqrt{3}a,$ and in the *CD* direction 3a (Figure 6b), in which only the double cell walls of the regular hexagon core, marked in red in Figure 6, are taken into account.

In the case of core bending stiffness in the *CD* direction, a beam is bent with length a (the length of the side of the hexagonal cell), height *h* (the core height), thickness 2$g_{r}$ ($g_{r}$ is the thickness of paper used for core production), and Young’s modulus *E_rCD_* of the core paper in the *CD* direction.

The moment of inertia of the beam is as follows:

(13) $$J_{rCD}=\frac{{2g}_{r}h^{3}}{12}\;$$

The core bending stiffness in the *CD* direction is calculated from the formula:

(14) $${BS}_{r2}=\frac{{2g}_{r}h^{3}}{12}\;\frac{E_{rCD}b}{\sqrt{3}a}\frac{2a}{3a}=\frac{E_{rCD}g_{r}h^{3}b}{15.6a}$$

After dividing by the sample width *b*, we obtain:

(15) $${BS}_{rCD}=\frac{{BS}_{r2}}{b}=\frac{E_{rCD}g_{r}h^{3}}{15.6a}$$

In the case of core stiffness in the *MD* direction, the bending of a honeycomb wall with length *h*, width 2a, and thickness 2$g_{r}$ is considered, for which the moment of inertia is equal to the following:

(16) $$J_{rMD}=\frac{2a{\left(2g_{r}\right)}^{3}}{12}\;$$

The assumed width of the bent wall was 2a, because, within the *ACBE* periodic cell, three sections of lengths a/2, a, and a/2 are bent in the *MD* direction of the cellular paperboard, which gives 2a (Figure 6).

The following formula was proposed to calculate the core bending stiffness in the *MD* direction:

(17) $${BS}_{r1}=\frac{2a{\left(2g_{r}\right)}^{3}}{12}\frac{E_{rMD}b}{a}\frac{2a}{3a}\frac{h}{2g_{r}}=\frac{E_{rMD}g_{r}^{2}bh}{2.24}$$

After dividing by the sample width *b*, we obtain:

(18) $${BS}_{rMD}=\frac{{BS}_{r1}}{b}=\frac{E_{rMD}g_{r}^{2}h}{2.24}$$

#### 2.2.3. Bending Stiffness of Cellular Paperboard

The bending stiffness of the cellular paperboard, both in the machine *MD* and cross *CD* direction, was calculated as the sum of the stiffness of the flat layers and the cellular paperboard core:

(19) $${BS}_{MD}={BS}_{oMD}+{BS}_{rMD}$$

(20) $${BS}_{CD}={BS}_{oCD}+{BS}_{rCD}$$

From (7) and (18), we obtain the bending stiffnesses of cellular paperboard in the machine direction *BS_MD_*:

(21) $${BS}_{MD}=D_{11}\left[1-\frac{B_{11}^{2}}{A_{11}D_{11}}\right]+\frac{E_{rMD}g_{r}^{2}h}{2.24}$$

From (12) and (15), we obtain the bending stiffness of cellular paperboard in the cross direction *BS_CD_*:

(22) $${BS}_{CD}=D_{22}\left[1-\frac{B_{22}^{2}}{A_{22}D_{22}}\right]+\frac{E_{rCD}g_{r}h^{3}}{15.6a}$$

## 3. Results and Discussion

Table 3 includes the results of measurements of the paper’s physical properties performed within this work. Table 4 and Table 5 present the results of measurements of paperboard thickness and bending stiffness.

**Figure 7:**
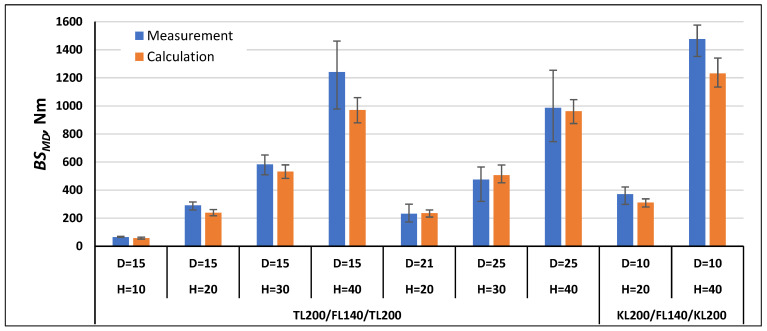
Results of *BS_MD_* measurements and calculations for cellular paperboards TL200/FL140/TL200 and KL200/FL140/KL200.

**Figure 8:**
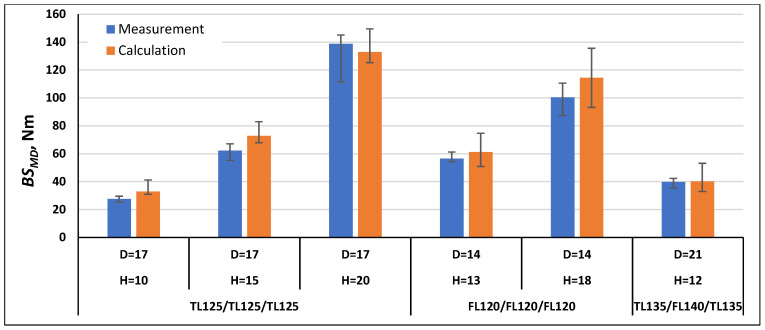
Results of *BS_MD_* measurements and calculations for cellular paperboards TL125/TL125/TL125, FL120/FL120/FL120, and TL135/FL140/TL135.

**Figure 9:**
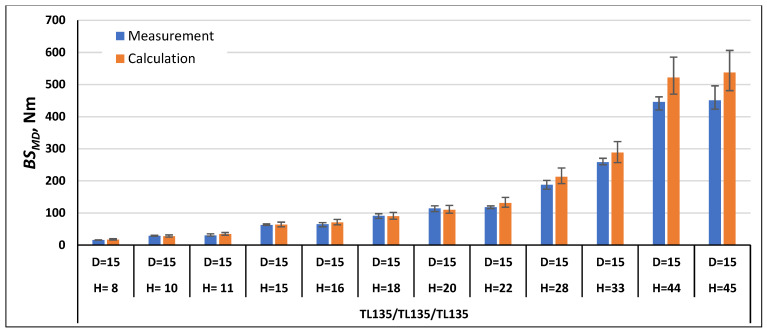
Results of *BS_MD_* measurements and calculations for cellular paperboards TL135/TL135/TL135.

**Figure 10:**
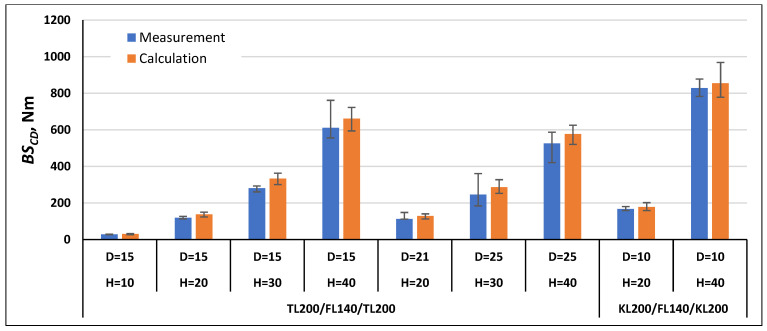
Results of *BS_CD_* measurements and calculations for cellular paperboards TL200/FL140/TL200 and KL200/FL140/KL200.

**Figure 11:**
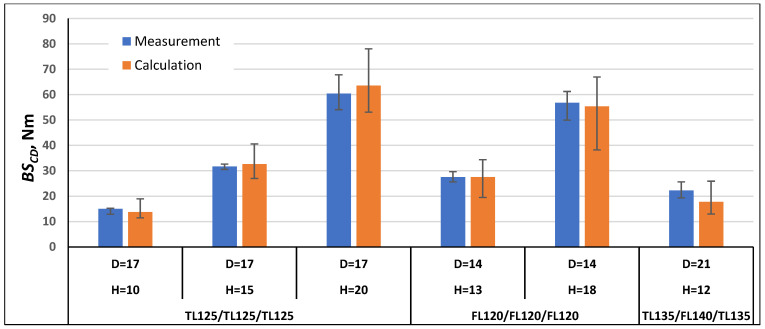
Results of *BS_CD_* measurements and calculations for cellular paperboards TL125/TL125/TL125, FL120/FL120/FL120, and TL135/FL140/TL135.

**Figure 12:**
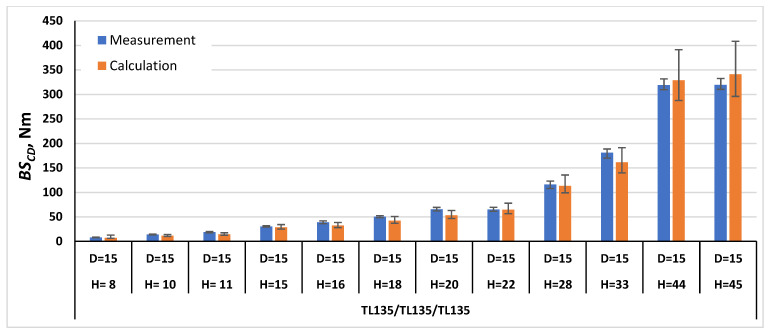
Results of *BS_CD_* measurements and calculations for cellular paperboards TL135/TL135/TL135.

In order to validate the calculation method using Formulas (21) and (22), the bending stiffness in the machine direction *BS_MD_* and cross direction *BS_CD_* were calculated for the paperboards, which were examined as part of this work, and for the paperboards marked with the symbols TL200/FL140/TL200 and TL135/TL135/TL135 tested as part of the work [1]. The comparison of the measurement results with the calculation results is shown in Figure 7, Figure 8, Figure 9, Figure 10, Figure 11 and Figure 12. The error bars for the measured *BS* values represent the maximum and minimum values obtained by measurements and, in the case of the calculated *BS* value, they represent the values calculated using the maximum and minimum values obtained by measuring the values of the physical properties of the paper raw materials used to produce paperboard and the geometric parameters of honeycomb paperboard.

**Figure 13:**
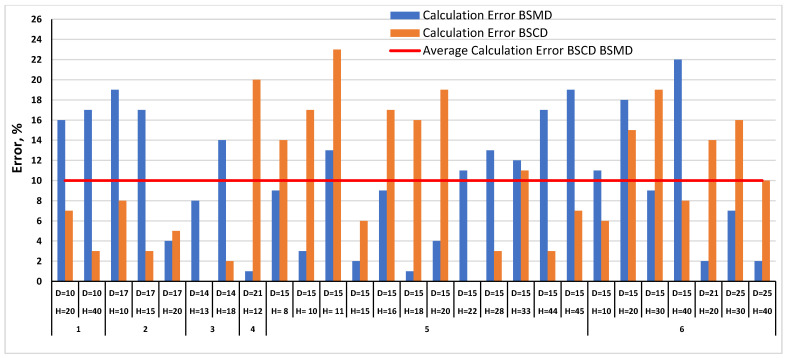
Values of *BS_MD_* and *BS_CD_* calculation errors and average error: 1—KL200/FL140/KL200, 2—TL125/TL125/TL125, 3—FL120/FL120/FL120, 4—TL135/FL140/TL135, 5—TL135/TL135/TL135, 6—TL200/FL140/TL200.

Figure 13 shows the calculation error obtained by using the *BS_MD_* and *BS_CD_* calculation methods proposed in this work. The calculation error was defined as an absolute value of the difference between the measurement and calculation results, divided by the measurement value rounded to the integer value, and expressed as a percentage.

The calculated *BS_MD_* values for paperboards with the raw material composition TL200/FL140/TL200 and KL200/FL140/KL200 (Figure 7) in most paperboards were lower than the measurement values, and the calculation error was within the range from 2% to 22% in relation to the values obtained during measurements. Only for two paperboards with the raw material composition TL200/FL140/TL200, thickness *H* = 20 mm, and mesh diameter *D* = 21 mm, as well as *H* = 30 mm and *D* = 25 mm, were they slightly higher by 2% and 7%, respectively, compared to the values obtained during measurements.

The calculated *BS_MD_* values for paperboards with the raw material composition TL125/TL125/TL125, FL120/FL120/FL120, and TL135/FL140/TL135 (Figure 8) in most paperboards were higher than the measurement values, and the calculation error was within the range from 1% to 19% in relation to the values obtained during measurements. Only for one paperboard, with the raw material composition TL125/TL125/TL125, mesh diameter *D* = 17 mm, and *H* = 20 mm, was the calculation value lower by 4% than the values obtained during measurements.

In the case of paperboard with the raw material composition TL135/TL135/TL135 (Figure 9), for most paperboards, the *BS_MD_* calculation results were higher than the measurement results and the difference was within the range from 2% to 19% in relation to the value obtained during measurements. In the case of three paperboards with *D* = 15 mm and *H* = 10, 18, and 20 mm, the calculated values were lower than the measured values by 3%, 1%, and 4%, respectively.

The calculated *BS_CD_* values for paperboards with the raw material composition TL200/FL140/TL200 and KL200/FL140/KL200 (Figure 10) for all tested paperboards were higher than the measurement values, and the difference was within the range from 3% to 19% in relation to the value obtained during measurements.

The calculated *BS_CD_* values for paperboard with raw material composition TL125/TL125/TL125, FL120/FL120/FL120, and TL135/FL140/TL135 (Figure 11) were close to the measurement values; the calculation error was within the range from 0% to 20%. For paperboard with raw material composition FL120/FL120/FL120, *D* = 14 mm, and *H* = 13 mm, the calculated value was equal to the measured value.

In the case of paperboards with the raw material composition TL135/TL135/TL135 (Figure 12), the *BS_CD_* calculation results for most paperboards were lower than the measurement results, and the difference was within the range from 0% to 23% in relation to the value obtained during measurements. Only for two paperboards, with *D* = 15 mm and *H* = 44, 45 mm, were the calculation values higher than the measurement values by 3% and 7%, respectively, in relation to the value obtained by measurements.

The calculation results and errors are included in Appendix B in Table A1 for all paperboards with different raw material compositions and geometric parameters. The maximum two errors are less than 25%: one is equal to 20% and the rest are less than 20%. The average error values for the *MD* and *CD* directions are the same and amount to 10%. It should be noted that the proposed patterns are based on an ideal hexagonal honeycomb structure and not on actual structures. This makes it possible to state that the authors consider the presented method to be consistent with experimental studies.

## 4. Summary and Conclusions

The proposed method makes it possible to calculate the bending stiffness *BS* of cellular honeycomb paperboard in the machine direction *MD* and the cross direction *CD,* based on the paperboard geometric parameters and the physical properties of the materials used for its production. It is much easier and faster in practical application than the popular FEM numerical methods. The developed model takes into account the ideal honeycomb structure, which does not always reflect the actual structure, and the buckling of the flat layers subjected to compression during bending stiffness measurement. The obtained calculation results gave a much more accurate representation of the measurement results compared to the results obtained by means of the method presented in the article [1], where the average measurement error in the machine direction *MD* for the tested group of paperboards was approximately 65% of the measured value and in the cross direction *CD* 31%.

In the tested range of paperboards with different raw material compositions and different geometric parameters in each of the main directions in the paperboard plane, theoretically calculated values differed from the real values by an average of 10% of the real value in both the machine *MD* and cross direction *CD*. The proposed calculation method provided a much more accurate representation of the measurement results compared to the method presented in the article [1]. Owing to the possibility of *BS* forecasting, prior to producing paperboard with the required stiffness, it is possible to select appropriate the fibrous raw materials and geometric parameters of the paperboard so that the product meets the customer’s requirements and is economical in production.

## Figures and Tables

**Table 1:** Raw material composition of cellular paperboards.

Paperboard Designation	Raw Material of Flat Layers	Weight (g/m^2^)	Paper Designation	Core Raw Material	Weight (g/m^2^)	Paper Designation
KL200/FL140/KL200	Krafliner	200	KL200	Fluting	140	FL140
TL125/TL125/TL125	Testliner	125	TL125	Testliner	125	TL125
FL120/FL120/FL120	Fluting	120	FL120	Fluting	120	FL120
TL135/FL140/TL135	Testliner	135	TL135	Fluting	140	1FL140

**Table 2:** Geometric parameters of cellular paperboards.

Paperboard Designation	*D* (mm)	*Hp* (mm)
KL200/FL140/KL200	10	20
		40
TL125/TL125/TL125	17	10
		15
		20
FL120/FL120/FL120	14	13
		18
TL135/FL140/TL135	21	12

*Hp*—paperboard thickness given by the manufacturer.

**Table 3:** Results of the measurements of paper physical properties.

Paper Designation	Paper Thickness (mm)	Max. Paper Thickness (mm)	Min. Paper Thickness (mm)	*E_CD_* (GPa)	*E_CD Max_* (GPa)	*E_CD Min_* (GPa)	*E_MD_* (GPa)	*E_MD Max_* (GPa)	*E_MD Min_* (GPa)
KL200	0.290	0.301	0.281	2.520	2.730	2.420	5.490	5.650	5.290
TL125	0.175	0.185	0.171	1.592	1.812	1.392	5.021	5.242	4.943
FL120	0.170	0.186	0.153	1.782	1.872	1.469	5.640	5.832	5.454
TL135	0.202	0.232	0.187	1.530	1.762	1.330	4.390	4.639	4.174
1FL140	0.213	0.252	0.198	1.421	1.774	1.034	3.881	4.080	3.556

*E_CD_*, *E_MD_*—average values of Young’s modules of paper in cross and machine direction obtained during measurements; *E_CD Max_*, *E_MD Max_*—maximum values of Young’s modules of paper in cross and machine direction obtained during measurements; *E_CD Min_*, *E_MD Min_*—minimum values of Young’s modules of paper in cross and machine direction obtained during measurements.

**Table 4:** Results of measurements of paperboard thickness *H*.

Paperboard Designation	*D* (mm)	*Hp* (mm)	*H* (mm)	*H_Max_* (mm)	*H_Min_* (mm)
KL200/FL140/KL200	10	20	20.02	20.15	19.62
		40	39.56	39.93	39.28
TL125/TL125/TL125	17	10	10.05	10.70	9.94
		15	14.92	15.18	14.70
		20	20.14	20.36	19.94
FL120/FL120/FL120	14	13	13.11	13.63	12.81
		18	17.91	18.33	17.32
TL135/FL140/TL135	21	12	11.74	12.24	11.32

*H*—measured paperboard thickness. *H_Max_*—maximum paperboard thickness obtained during measurements. *H_Min_*—minimum paperboard thickness obtained during measurements.

**Table 5:** Results of *BS_MD_* and *BS_CD_* measurements.

Paperboard Designation	*D* (mm)	*H* (mm)	*BS_MD_* (Nm)	*BS_MD Max_* (Nm)	*BS_MD Min_* (Nm)	*BS_CD_* (Nm)	*BS_CD Max_* (Nm)	*BS_CD Min_* (Nm)
KL200/FL140/KL200	10	20.02	372	423	298	166	181	158
		39.56	1476	1576	1353	828	878	782
TL125/TL125/TL125	17	10.05	28	30	25	15	15	13
		14.92	62	67	55	32	33	31
		20.14	139	145	111	60	68	54
FL120/FL120/FL120	14	13.11	57	61	54	28	30	26
		17.91	100	111	87	57	61	50
TL135/FL140/TL135	21	11.74	40	42	35	22	26	19

*BS_MD Max_*, *BS_CD Max_*—maximum value of bending stiffness obtained by measurements in the *MD* and *CD* directions, respectively; *BS_MD Min_*, *BS_CD Min_*—minimum value of bending stiffness obtained by measurements in the *MD* and *CD* directions, respectively.

## Data Availability

Data are contained within the article.

## Appendix A

In the proposed *BS* calculation model, the classical laminated plate theory (CLPT) 2D formulas [32,33] were used as adapted to the 1D beam model. This means that the Poisson number in each layer of cellular cardboard was disregarded in the formulas.

According to the classical laminated plate theory (CLPT), the elements of the single *k*-th stiffness matrix of three layers for the 1D model are determined from the relationship:

(A1) $${\left(Q_{11}\right)}_{k}=\frac{E_{1k}}{1-\nu_{12}\nu_{21}}\approx E_{1k},{\left(Q_{22}\right)}_{k}=\frac{E_{2k}}{1-\nu_{12}\nu_{21}}\approx E_{2k}$$

The arrangement diagram of individual layers of cellular paperboard is shown in Figure A1. It was assumed that the thickness of the three layers of paperboard, i.e., the total thickness *H* of the cellular paperboard, is equal to the sum of the thickness of the two covering layers and the height of the cellular core.

(A2) $$H=g_{1}+h+g_{3}$$

In the cellular paperboard being considered, the flat layers are made of the same paper, i.e., $g_{1}=g_{3},$ and the height of the cellular core is marked as *h*. The number of cardboard layers is k = 3 (Figure A1). As can be seen, an index marking of individual paperboard layers has been applied. Moreover, *z_o_* = –*H*/2 and *z_k_* = *z*_3_ = *H*/2.

The stiffness matrix coefficients of each layer for the 2D plate model in accordance with CLPT [32,33], which are constant for a given layer, can be written as follows:

(A3) $$A_{ij}=\sum_{k=1}^{3}{\left(Q_{ij}\right)}_{k}(z_{k}-z_{k-1})$$

(A4) $$B_{ij}=\frac{1}{2}\sum_{k=1}^{3}{\left(Q_{ij}\right)}_{k}(z_{k}^{2}-z_{k-1}^{2})$$

(A5) $$D_{ij}=\frac{1}{3}\sum_{k=1}^{3}{\left(Q_{ij}\right)}_{k}(z_{k}^{3}-z_{k-1}^{3})$$

Taking into account relationships (A3)–(A5), according to CLPT, the resultant forces and cross-sectional moments acting in cellular paperboard have the form:

(A6) $$\left\{\begin{array}{l} \left\{\;N\;\right\}\\ \left\{\;M\;\right\} \end{array}\right\}=\left[\begin{array}{cc} \left[A\right] & \left[B\right]\\ \left[B\right] & \left[D\right] \end{array}\right]\left\{\begin{array}{l} \left\{\varepsilon \right\}\\ \left\{\kappa \right\} \end{array}\right\}=\left[K\right]\left\{\begin{array}{l} \left\{\varepsilon \right\}\\ \left\{\kappa \right\} \end{array}\right\}$$

The matrix [*A*], defined by the relationship (A3) is called the tensile stiffness; the matrix [*D*] expressed by the equation (A5), corresponding to the bending of the laminate from the mid-plane, is called the flexural stiffness; and the matrix [*B*] described by the formula (A4) resulting from the coupling between forces {*N*} and moments {*M*} or between deformations {*ε*} and curvatures {*κ*}, is called coupling (or interaction) stiffness. It should be noted that in plate theory, cross-sectional forces and moments are related to the plate length unit. A similar situation applies to the elements of the stiffness matrix [*A*], [*B*], and [*D*]. The above elements of plate stiffness can be interpreted as the stiffness of cellular paperboard. In this work, it was assumed that direction 1 corresponds to the machine direction *MD*, and direction 2—to the cross direction *CD*.

In the case of a laminated beam element (i.e., 1D model) in direction 1 (*MD*), relationships (A6) take the form:

(A7) N¯1=N1b1=(A11ε1+B11κ1)b1M¯1=M1b1=(B11ε1+D11κ)1b1

Hence, for direction 2 (*CD*), similarly to (A7), we obtain the following relationships:

(A8) N¯2=N2b2=(A22ε2+B22κ2)b2M¯2=M2b2=(B22ε2+D22κ)2b2

In relationships (A7) and (A8), the values *b*_1_ and *b*_2_ denote the widths of beam elements in the CD and *MD* directions.

In order to determine the bending stiffness of cellular paperboard using the four-point method in direction 1 (*MD*), it was assumed that ${\bar{N}}_{1}=0,\;{\bar{M}}_{1}\neq 0$; that is, only the load is bending. Then, taking this assumption into account, formula (A7) takes the form:

(A9) $${\bar{M}}_{1}=(D_{11}-B_{11}^{2}/A_{11})b_{1}\kappa_{1}=D_{11}[1-B_{11}^{2}/(A_{11}D_{11})]b_{1}\kappa_{1}$$

After introducing the reduced stiffness coefficient $\alpha_{1}$, which is defined as follows:

(A10) $$\alpha_{1}=[1-B_{11}^{2}/(A_{11}D_{11})]$$

the relationship (A9) finally takes the form:

(A11) $${\bar{M}}_{1}=D_{11}\alpha_{1}b_{1}\kappa_{1}$$

Stiffness for cellular board under four-point bending in machine direction 1 (*MD*) interpreted in accordance with (A11) as a linear relationship between the bending moment $\bar{M_{1}}$ and the curvature $\kappa_{1}$ is described by the following relationship:

(A12) $${BS}_{1}=\frac{\bar{M_{1}}}{\kappa_{1}}=D_{11}\alpha_{1}b_{1}=D_{11}^{*}b_{1}$$

where

$D_{11}^{*}$= $D_{11}\alpha_{1}$—effective plate stiffness in the machine direction.

In order to determine the stiffness of cellular paperboard in direction 2 (*CD*) with four-point bending, we proceed in the same way as for direction 1 (*MD*). Assuming that ${\bar{N}}_{2}=0,\;{\bar{M}}_{2}\neq 0,$ and introducing, similarly, to the (A8) designation of the reduced coefficient $\alpha_{2}$ defined by the equation:

(A13) $$\alpha_{2}=[1-B_{22}^{2}/(A_{22}D_{22})]$$

we obtain:

(A14) $${\bar{M}}_{2}=D_{22}\alpha_{2}b_{2}\kappa_{2}$$

The bending stiffness in direction 2 (*CD*) of cellular paperboard is determined from the following relationship:

(A15) $${BS}_{2}=\frac{\bar{M_{2}}}{\kappa_{2}}=D_{22}\alpha_{2}b_{2}=D_{22}^{*}b_{2}$$

where $D_{22}^{*}$= $D_{22}\alpha_{2}$—effective plate stiffness in the cross direction.

Since the *BS*_1_, *BS*_2_ indicators are related to the unit length, the dimensions *b*_1_ and *b*_2_ can be assumed as unit dimensions and *b*_1_ = *b*_2_ = *b* = 100 mm.

## Appendix B

**Table A1:** Results of *BS_MD_* and *BS_CD_* calculations and calculation error.

Paperboard Designation	*D* (mm)	*H* (mm)	Calculation *BS_MD_* (Nm)	Calculation Error *BS_MD_* (%)	Calculation *BS_CD_* (Nm)	Calculation Error *BS_CD_* (%)
KL200/FL140/KL200	10	20	312	16	178	7
		40	1231	17	854	3
TL125/TL125/TL125	17	10	33	19	14	8
		15	73	17	33	3
		20	133	4	64	5
FL120/FL120/FL120	14	13	61	8	28	0
		18	114	14	55	2
TL135/FL140/TL135	21	12	40	1	18	20
TL135/TL135/TL135	15	8	17	9	7	14
		10	28	3	12	17
		11	34	13	15	23
		15	64	2	29	6
		16	71	9	32	17
		18	90	1	42	16
		20	110	4	53	19
		22	131	11	65	0
		28	213	13	113	3
		33	288	12	161	11
		44	522	17	329	3
		45	538	19	341	7
TL200/FL140/TL200	15	10	58	11	30	6
		20	239	18	137	15
		30	532	9	333	19
		40	971	22	661	8
	21	20	236	2	128	14
	25	30	507	7	286	16
		40	963	2	576	10
			**Average Calculation Error *BS_MD_*** **(%)**	10	**Average Calculation Error *BS_CD_*** **(%)**	10
